# Phase IV noninferiority controlled randomized trial to evaluate the impact on diagnostic thinking and patient management and the test–retest reproducibility of the Gaxilose test for hypolactasia diagnosis

**DOI:** 10.1097/MD.0000000000013136

**Published:** 2018-11-16

**Authors:** Carmen Monsalve-Hernando, Laura Crespo, Blanca Ferreiro, Verónica Martín, Xavier Aldeguer, Verónica Opio, Pedro Luis Fernández-Gil, María Jesús Gaspar, Eduardo Romero, Carmen Lara, Cecilio Santander, Leyanira Torrealba, Theodora Savescu, Carmen Hermida

**Affiliations:** a Venter Pharma S.L. Alcobendas, Madrid; b Gastroenterology Service, Hospital Universitario Ramón y Cajal, Madrid; c Digestive System Service, Hospital Universitario Virgen de la Victoria, Málaga; d Digestive System Service, Hospital Universitario de la Princesa, Madrid; e Digestive System Service, Hospital Universitario Doctor Josep Trueta, Gerona; f Digestive System Service, Hospital Universitario de Getafe, Getafe, Madrid; g Digestive System Service, Hospital Universitario Marqués de Valdecilla, Santander, Cantabria; h Clinical Analysis Laboratory, Hospital Universitario de Getafe, Getafe, Madrid, Spain..

**Keywords:** diagnostic thinking, gaxilose, hypolactasia, patient management, reproducibility

## Abstract

**Background:**

The diagnostic accuracy of the Gaxilose test (GT) for hypolactasia diagnosis has already been proved. The objectives of this clinical trial were to demonstrate the noninferiority of the GT compared to the hydrogen breath test (HBT) on the impact on diagnostic thinking and patient management, to evaluate the GT reproducibility with urine accumulated from 0 to 4 hours and from 0 to 5 hours and to assess test safety.

**Methods:**

We conducted a randomized, parallel, noninferiority clinical trial. Patients with clinical symptoms suggestive of lactose intolerance were screened for inclusion and randomly assigned to the GT arm or the HBT arm of the study. The impact on diagnostic thinking and patient management was analyzed with pretest and posttest questionnaires in which the investigators indicated their estimated probability of hypolactasia diagnosis and the intended management before and after the GT or the HBT (noninferiority margin: −10%). The primary outcome of the study was the impact on diagnostic thinking, expressed as the mean of the absolute values of the differences between the pretest and posttest probabilities of hypolactasia diagnosis. Patients randomized to the GT arm performed also the retest to evaluate the reproducibility of the GT.

**Results:**

A total of 147 patients were included in the intend-to-treat (ITT) population. Among them, 74 performed the HBT and 73 performed the GT. The results proved the noninferiority of the GT compared to the HBT on the impact on diagnostic thinking (Impact_GT_ = 31.74 ± 23.30%; Impact_HBT_ = 24.28 ± 19.87%; Δ_GT−HBT_ = 7.46%; 95% confidence interval of Δ_GT−HBT_: 1.55%, infinite) and on patient management. The test–retest reproducibility was better for the GT with urine accumulated from 0 to 5 h: the intraclass correlation coefficient (ICC) was 0.5761, and the Kappa coefficient was 0.7548, indicative of substantial agreement between both tests. No serious adverse events were reported during the study.

**Conclusions:**

The GT has an impact on diagnostic thinking and patient management noninferior to that of the HBT, is reproducible and well tolerated. These results prove the clinical benefit of its use in the clinical practice (ClinicalTrials.gov identifier: NCT02636413).

## Abstract 2

Supplemental Digital Content is available in the text

## 1 Introduction

More than half of the world population develop nonspecific symptoms of lactose intolerance such as abdominal distension and pain, bloating, and diarrhea after consumption of dairy products. This symptomatic response is, in general, a consequence of the deficiency in intestinal lactase, also known as hypolactasia, which leads to an inefficient digestion of lactose.^[1–3]^

The Gaxilose test (GT) is a new noninvasive diagnostic method for hypolactasia based on the oral administration of Gaxilose (4-*O*-β-d-galactopyranosyl-d-xylose), a synthetic disaccharide that is a close structural analogue of lactose. Gaxilose is hydrolyzed by intestinal lactase into 2 physiological products: galactose and xylose.^[4,5]^ Xylose is passively absorbed^[6]^ and partially metabolized, with the rest appearing in blood and being finally excreted in urine. Total xylose amount in urine as well as xylose concentration in blood represent a measure of total lactase activity *in vivo.*^[4,7,8]^

An extensive preclinical and clinical development program was carried out for the GT with urine accumulated from 0 to 4 hours and from 0 to 5 hours and the Gaxilose blood test.^[4,7,8]^ It included the performance of 3 clinical trials, which demonstrated the tolerability and diagnostic accuracy of these tests, with excellent specificity, sensitivity and safety, higher than those of the hydrogen breath test (HBT) and lactose tolerance test, commonly used in the clinical practice.^[1–3]^ The GT with urine accumulated from 0 to 5 hours has been in fact commercialized in Spain since 2013.

However, many authors have insisted that the diagnostic accuracy is not sufficient to evaluate a new diagnostic test because its clinical benefit relies on its positive influence in patient management and well-being.^[9–12]^ As it is stated in the European Medicines Agency (EMA) guideline on Clinical Evaluation of Diagnostic Agents,^[13]^ the clinical utility of a diagnostic agent depends on whether it can decrease the diagnostic uncertainty and guide patient management. Performance of a prospective randomized clinical trial is the recommended strategy to evaluate the impact on diagnostic thinking and patient management of a new test in comparison with that of a preexisting commonly used test.^[9,11]^

Therefore, the primary and the first secondary objective of this clinical trial were to demonstrate the noninferiority of the GT compared to the HBT on the impact on diagnostic thinking and patient management, respectively, for the diagnosis of hypolactasia. The HBT was selected as active comparator because it is the most frequently used diagnostic test for hypolactasia in the current practice.^[2,14]^ The rationale behind the use of a noninferiority design was based on the absence to our knowledge of previous studies about the impact on diagnostic thinking and patient management of the HBT, as well as the subjective nature of the evaluated parameters.^[9,12]^ As the GT has been proved to have higher diagnostic performance and fewer side effects than the HBT,^[8]^ noninferiority on the impact on diagnostic thinking and patient management was a suitable result. Additional secondary objectives were to demonstrate GT reproducibility with urine accumulated from 0 to 4 hours and from 0 to 5 hours after Gaxilose administration, since it had not been assessed previously. Finally, the safety of both diagnostic procedures was also evaluated.

## 2 Methods

### 2.1 Patients

Patients of either sex aged 18 to 70 years who were able to understand and give the informed consent, presented clinical symptoms suggestive of lactose intolerance, had not been diagnosed yet, and fulfilled the requirements of the GT summary of product characteristics (SmPC)^[15]^ were enrolled in this study by the investigators in the Gastroenterology Service of 6 Spanish hospitals. Exclusion criteria included inability or reticence to give the informed consent or to comply with the study requirements, pregnancy or lactation, abnormal glomerular filtration rate, portal hypertension, medical records of total gastrostomy and/or vagotomy, myxedema, diabetes mellitus, participation in another clinical trial during the 3 months prior to inclusion in this study, drug abuse, treatment with antibiotics and antiparasitics 7 days prior to the HBT, consumption of aspirin or indomethacin in the 48 hours preceding the GT, or any disorder that might interfere with any of the diagnostic tests.

### 2.2 Design and randomization

This study was a phase IV, multicentric, randomized, parallel, open-label, noninferiority clinical trial, with the HBT as active comparator. After assessment of the inclusion and exclusion criteria, each patient was randomly assigned to the GT arm or the HBT arm of the study. Allocation of the tests was performed in a 1:1 ratio stratified per center and blocked by 4 using a centralized online randomization system. The randomization was performed with the RERAND module of the electronic data capture software remote data capture (RDC) Onsite of Oracle, version 5.1 (Oracle Corporation, Redwood City, CA).

The study was conducted in accordance with the Declaration of Helsinki and with Good Clinical Practice guidelines, and was approved by the Ethics Committee for Investigation with medicinal products of the study (Ethics Committee for Investigation with Medicinal Products of the Hospital Universitario Ramón y Cajal, Madrid, Spain) and by the Ethics Committees of all the participating centers. All the patients provided written informed consent for their participation in the study.

### 2.3 Gaxilose test procedure

The test was performed in the 3 days following the patient's randomization. Patients had to fast for 10 hours and empty their bladder immediately before the test. Then, 0.45 g of Gaxilose were orally administered to the patients dissolved in 100 mL of water. Total urine excreted from 0 to 4 hours and from 4 to 5 hours after Gaxilose administration was collected in 2 different containers. Patients allocated to the GT arm had also to perform the Gaxilose retest. For that purpose, they repeated the same procedure with a washout period of 5 ± 2 days.

Total urine volume collected in each container was measured and 3 aliquots with 1.5 mL of urine from each container were stored at −20°C in each center. Among each set of 3 aliquots, 2 were transported to the study central laboratory (Clinical Analysis Laboratory of the Hospital Universitario de Getafe) frozen in dry ice. There, xylose quantification in 0 to 4 hours and 4 to 5 hours urine samples was conducted by the phloroglucinol colorimetric reaction described in the GT SmPC.^[15,16]^ A total xylose amount in the urine collected from 0 to 5 hours after Gaxilose administration lower than 37.87 mg was indicative of hypolactasia diagnosis.

### 2.4 Hydrogen breath test procedure

Patients allocated to the arm of the HBT performed the test only once, in the 3 days following the patient's randomization. Dosage and measurement procedure were selected according to the site clinical protocol. A lactose dose comprised between 25 and 50 g dissolved in 250 to 500 mL of water was orally administered to the patient after fasting for 12 hours. The amount of hydrogen in the expired air was measured before lactose administration and at different times after the lactose overload. An increment of more than 20 ppm in the hydrogen amount with respect to the basal value was indicative of hypolactasia.

### 2.5 Impact on diagnostic thinking

The impact on diagnostic thinking of both tests was assessed with a pretest and posttest visual analogue scale (VAS) questionnaire where the physician had to indicate his/her estimated probability for hypolactasia diagnosis before and after the performance of the HBT or the GT, being 0 “very improbable” and 100 “definitely probable.” The pretest questionnaire was completed during the screening visit and the posttest questionnaire during the last visit of the trial in which the physician informed the patient about the result of the diagnostic test (1 month later). To avoid biases, the pretest VAS questionnaire was not available for completion of the posttest VAS questionnaire. Moreover, for the GT arm, only the result of the first GT was known for completion of the posttest VAS questionnaire.

For each patient, the absolute value of the difference between the score of the pretest and posttest VAS questionnaires was calculated. This difference represented the impact on diagnostic thinking and the primary variable of the trial. The values for the GT and the HBT were later compared between them.

### 2.6 Impact on patient management

**Figure 1:**
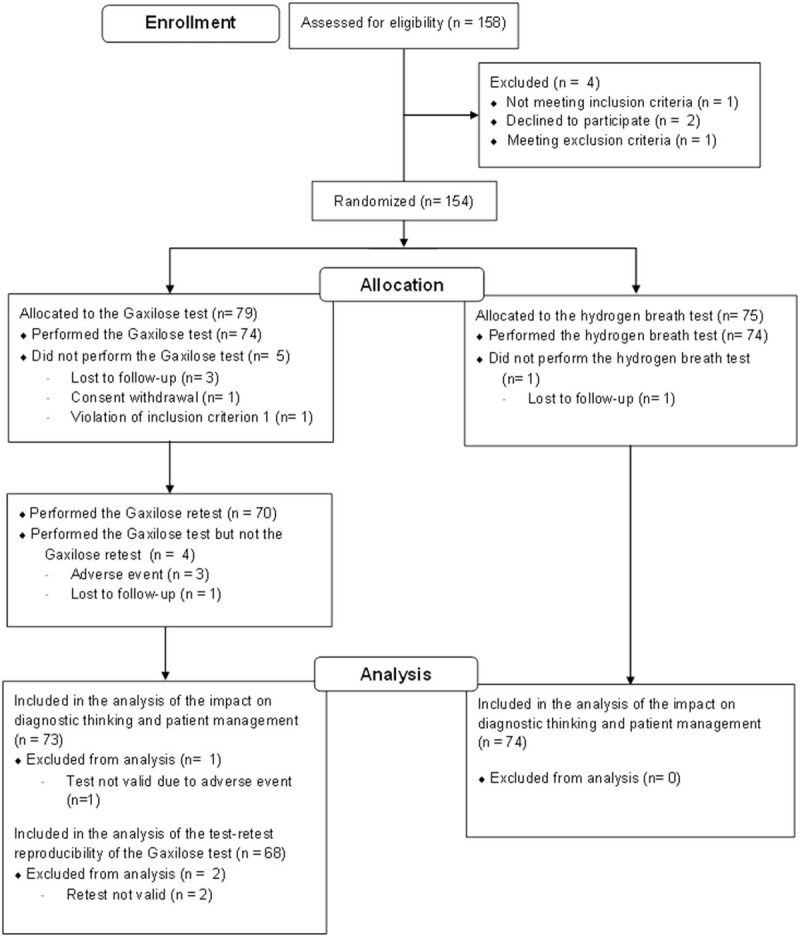
Flow diagram presenting the disposition of patients during the clinical trial.

The impact on patient management was evaluated with the pretest and posttest management questionnaires in which the investigator had to indicate his/her expected therapy before and after the performance of the hypolactasia diagnostic test, respectively. The options represented all the clinical available possibilities of patient management (see Figure 1, Supplemental Digital Content, for an example of management questionnaire used during the clinical trial). The pretest questionnaire was completed during the screening visit and the posttest questionnaire during the last visit of the trial. To avoid biases, the pretest management questionnaire was not available to complete the posttest questionnaire.

The impact on patient management was evaluated through the percentage of patients who passed from “*No intervention*” or “*Prescription of additional diagnostic tests*” or “*Referral to another specialist*” at pretest to “*Diet adjustment and follow-up”* at posttest for each one of the diagnostic tests.

### 2.7 Test–retest reproducibility of the Gaxilose test with urine accumulated from 0 to 4 hours

The total xylose amount excreted in the urine collected from 0 to 4 hours of the Gaxilose test and retest was quantified by the phloroglucinol method as previously reported.^[15,16]^ The reproducibility was evaluated through the intraclass correlation coefficient (ICC) and through the agreement Kappa coefficient,^[17–19]^ taking into account that the cut-off of the total xylose amount in urine accumulated from 0 to 4 hours is 27.58 mg.^[7,8]^ Therefore, patients with a xylose amount lower than this value would be considered hypolactasic.

### 2.8 Test–retest reproducibility of the Gaxilose test with urine accumulated from 0 to 5 hours

The total xylose amount excreted in the urine collected from 0 to 5 hours during the Gaxilose test and retest was calculated after quantifying the amount of monosaccharide in the 0 to 4 hours and 4 to 5 hours urine fractions. The reproducibility of the test was evaluated through the ICC and through the agreement Kappa coefficient.^[17–19]^ In this case, the cut-off of the total xylose amount in urine accumulated from 0 to 5 hours is 37.87 mg.^[7,8]^ Therefore, patients with a xylose amount lower than this value would be considered hypolactasic.

### 2.9 Safety assessment

Adverse events (AEs) were reported during the performance of the test and the 72 hours following it. The investigators performed a telephonic interview with the patient 6 ± 3 days after the Gaxilose retest or 7 ± 3 days after the HBT, to ensure the communication of any AE suffered by the patient after the performance of the test(s).

### 2.10 Statistics

The expected sample size was 144 patients (72 per arm) assuming 10% of losses or withdrawals. It was estimated that 64 analyzable patients per diagnostic test would be required to achieve ≥80% power to detect noninferiority in the impact on diagnostic thinking between the GT and the HBT, using a one-sided 2-sample *t* test. The limit of noninferiority for the lower limit of the 95% confidence interval (CI) of the difference was fixed to −0.10 (−10%), assuming that the true difference between the diagnostic tests was 0 and that the data were drawn from populations with standard deviations (SDs) of 0.20 and 0.20. The significance level (alpha) of the test was 0.025. This sample size would also allow to achieving at least 80% power to detect an ICC >0.6 under a Kappa coefficient estimation of 0.8.

The impact on diagnostic thinking and patient management was analyzed for all subjects in the intent-to-treat (ITT) and per protocol (PP) populations. The first one included all the randomized patients who performed the GT or the HBT and had at least one posttest assessment, and the second one included all subjects in the ITT population who completed the study without any major protocol violation. A noninferiority one-sided *t* test was used to compare the impact on diagnostic thinking between the tests. The margin of noninferiority was fixed to −0.10 that consists in a difference of −10%. A noninferiority 2-sided test based on the comparison of percentages through the binomial distribution approximation to the Gauss curve was used to compare the percentage of patients who passed from “*No intervention*” or “*Prescription of additional diagnostic tests*” or “*Referral to another specialist*” at pretest to “*Diet adjustment and follow-up”* at posttest for each one of the diagnostic tests. The margin of noninferiority was also fixed in −0.10 (−10%). GT reproducibility was analyzed for all subjects randomized to the GT arm who performed the test and retest (test–retest population); and safety data were analyzed for all subjects randomized who performed any of the diagnostic tests (safety population).

Data analysis, tabulations of descriptive statistics and inferential statistics were performed using SAS version 9.4 or higher and SAS Enterprise Guide version 7.1 (SAS institute, Cary, NC).

## 3 Results

### 3.1 Demographic and baseline characteristics

A total of 158 patients were screened for inclusion in this trial between October 2015 and October 2016. Among these, 154 patients were randomized, and 4 were excluded due to screening failure (Fig. 1). A total of 79 patients were randomly assigned to the GT arm of the study. Among them, 5 patients were withdrawn from the trial before performing the test and 74 performed the first GT. Four patients were withdrawn after the performance of the first GT but without performing the retest while 70 patients performed the Gaxilose retest. Finally, 2 patients withdrew their consent after performance of the Gaxilose retest, which was not valid due to the contamination of the collected urine samples. Regarding the other arm, 75 patients were randomized to the HBT arm. Among them, 74 performed the HBT, and only 1 patient was withdrawn from the study without performing the test. A total of 142 patients (92.21% of the randomized patients) completed the study procedures.

Table 1 summarizes the demographic and baseline characteristics of the recruited patients. No significant differences were observed between both arms of the ITT population. More women than men were recruited in the study (75%–80%) as reported in previous Gaxilose clinical studies.^[7,8]^ All the patients presented at least one symptom of lactose intolerance: abdominal distension, abdominal pain, diarrhea, flatulence, or other.

**Table 1:**
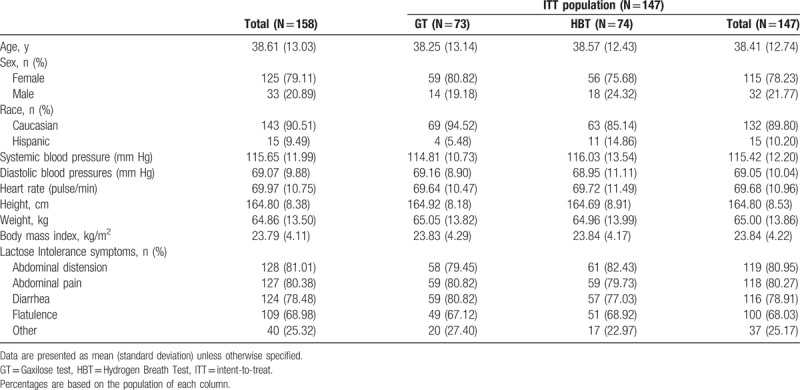
Demographic and baseline characteristics of the patients.

### 3.2 Impact on diagnostic thinking

The number of patients included in the ITT population was 147. Among those, 73 patients (49.66%) performed the GT, and 74 patients (50.34%) performed the HBT. Forty-two patients (28.57% of the ITT population) were diagnosed with hypolactasia and 105 patients (71.43% of the ITT population) were found to be normolactasic (Table 2).

**Table 2:**
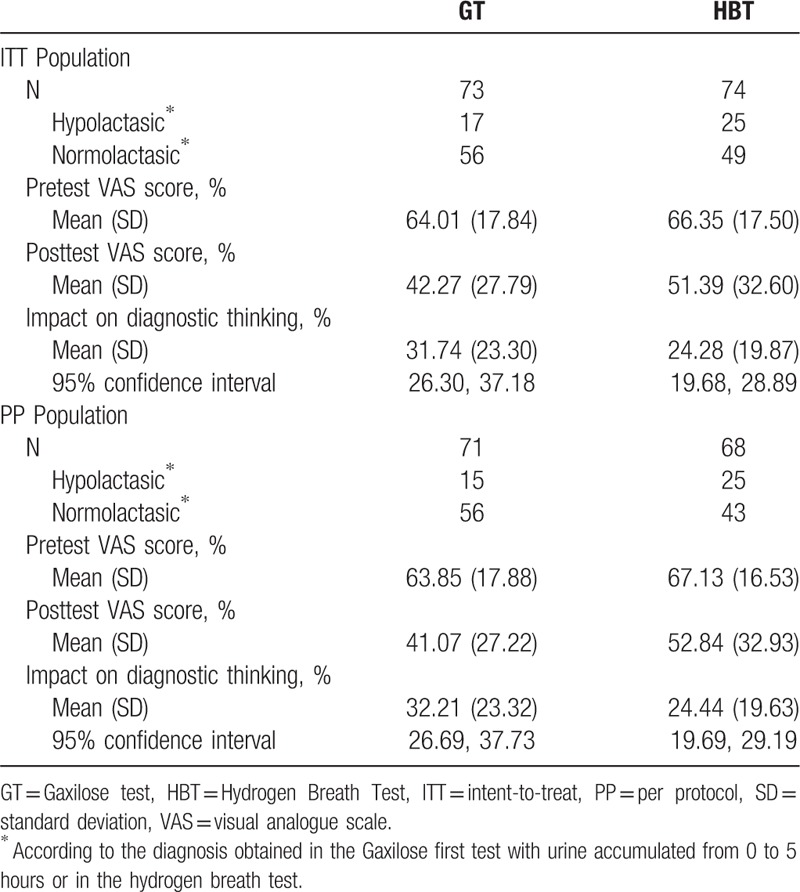
Results of the pretest and posttest visual analogue scale questionnaires and impact on diagnostic thinking, expressed as the absolute value of the difference between the pretest and posttest visual analogue scale scores.

The impact on diagnostic thinking expressed as mean (SD) of the absolute value of the differences between the pretest and posttest VAS scores was 31.74% (23.30) for the GT and 24.28% (19.87) for the HBT (Table 2). The difference between the impact of the GT and the HBT is 7.46% (95% CI: 1.55%, infinite). The limit of noninferiority was fixed in −10%. As the lower limit of the 95% CI of the difference is higher than this value, the noninferiority of the GT compared to the HBT on the impact on diagnostic thinking for the diagnosis of hypolactasia was demonstrated for the ITT population (*P* < .001).

In the case of the PP population, the number of included patients was 139. Among those, 71 patients (51.08%) performed the GT, and 68 patients (48.92%) performed the HBT. Forty patients (28.78% of the PP population) received an hypolactasia diagnosis and 99 patients (71.22% of the PP population) were found to be normolactasic (Table 2).

The impact on diagnostic thinking was 32.21% (23.32) for the GT and 24.44% (19.63) for the HBT (Table 2). The difference between the impact of the GT and the HBT is 7.77% (95% CI: 1.70%, infinite). As the lower limit of the 95% CI of the difference is higher than the limit of noninferiority (−10%), the noninferiority of the GT compared to the HBT on the impact on diagnostic thinking for the diagnosis of hypolactasia was also demonstrated for the PP population (*P* < .001).

### 3.3 Impact on patient management

The results of the pretest and posttest management questionnaires are summarized for the ITT population in Table 1, Supplemental Digital Content. The option most frequently indicated in the pretest management questionnaire was “*Diet adjustment and follow-up*” (117 patients, 79.59% of the ITT population), followed by the option “*No intervention*” (16 patients, 10.88%), “*Other diagnostic tests*” (11 patients, 7.48%), “*Diagnostic test for celiac disease*” (2 patients, 1.36%), and “*Diagnostic test for Crohn's disease*” (1 patient, 0.68%).

In the posttest management questionnaire, the number of patients whose indicated option was “*Diet adjustment and follow-up*” decreased to 66 (44.90% of the ITT population), whereas the number of patients with the option “*No intervention*” increased to 41 (27.89%), probably due to fact that only 42 patients (28.57%) of the ITT population were diagnosed with hypolactasia, whereas 105 patients (71.43%) of the ITT population were found to be normolactasic. Additionally, “*Other diagnostic tests*” was the option chosen for 31 patients (21.09%), probably also due to high number of negative diagnoses obtained. The other options marked were “*Diagnostic test for celiac disease*” (4 patients, 2.72%), “*Diagnostic test for Crohn's disease*” (2 patients, 1.36%), “*Referral to another specialist*” (2 patients, 1.36%), and “*Referral to a nutritionist*” (1 patient, 0.68%).

The impact on patient management was evaluated through the percentage of patients who passed from “*No intervention*” or “*Prescription of additional diagnostic tests*” or “*Referral to another specialist*” at pretest to “*Diet adjustment and follow-up*” at posttest. Only 5 patients (6.85%) of the 73 patients included in ITT population in the GT arm and 4 patients (5.41%) of the 74 patients included in ITT population in the HBT arm had management questionnaires according to this condition. The difference between the values of the GT and the HBT is 1.44% (95% CI: −6.31% to 9.20%). As the lower limit of the 95% CI is higher than the limit of noninferiority (−10%), the noninferiority of the GT compared to the HBT on the impact on patient management was demonstrated for the ITT population (*P* = .007).

The results of the pretest and posttest management questionnaires are summarized for the PP population in Table 2, Supplemental Digital Content. The frequency of the options marked was very similar to that found for the ITT population. The percentage of patients who passed from “*No intervention*” or “*Prescription of additional diagnostic tests*” or “*Referral to another specialist*” at pretest to “*Diet adjustment and follow-up*” at posttest was 5.63% for the GT (4 patients out of 71) and 5.88% for the HBT (4 patients among 68). The difference between the value of the GT and the value of the HBT is −0.25% (95% CI: −8.00 to 7.50). As the limit of noninferiority was fixed in −10%, and the lower limit of the 95% CI is higher than this value, the noninferiority of the GT compared to the HBT on the impact on patient management was also demonstrated for the PP population of the trial (*P* = .02).

### 3.4 Test–retest reproducibility of the Gaxilose test with urine accumulated from 0 to 4 hours

GT reproducibility was analyzed for the 68 subjects included in the test–retest population. The results of Gaxilose urine test and retest with urine accumulated from 0 to 4 hours are presented in Table 3. As it can be observed, 10 discrepancies were found between the 2 tests: 5 patients (7.35%) were classified as hypolactasic by the test and normolactasic by the retest and 5 patients (7.35%) were classified as normolactasic by the test and hypolactasic by the retest (see Table 3, Supplemental Digital Content, which indicates the xylose amounts obtained in each procedure for these patients). Taking into account those discrepancies, the Kappa coefficient was found to be 0.5913 (95% CI: 0.3639–0.8188). The obtained ICC was 0.5468.

**Table 3:**
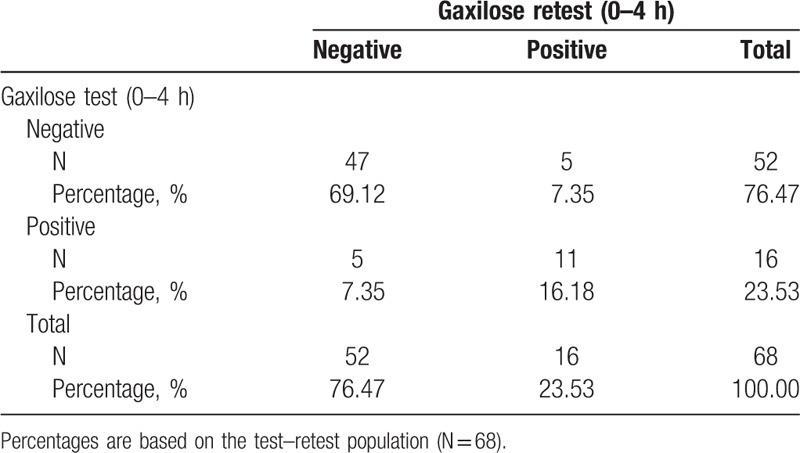
Results of the Gaxilose urine test and retest with urine accumulated from 0 to 4 hours (test-retest population).

### 3.5 Test–retest reproducibility of the Gaxilose test with urine accumulated from 0 to 5 hours

The results of Gaxilose test and retest with urine accumulated from 0 to 5 hours are presented in Table 4. In this case, 6 discrepancies were found between the 2 tests: 3 patients (4.41%) were classified as hypolactasic by the test and normolactasic by the retest and 3 patients (4.41%) were classified as normolactasic by the test and hypolactasic by the retest (see Table 4, Supplemental Digital Content, which presents the xylose amounts obtained in each procedure for these patients). The obtained Kappa coefficient was 0.7548 (95% CI: 0.5692–0.9404) and the ICC was 0.5761.

**Table 4:**
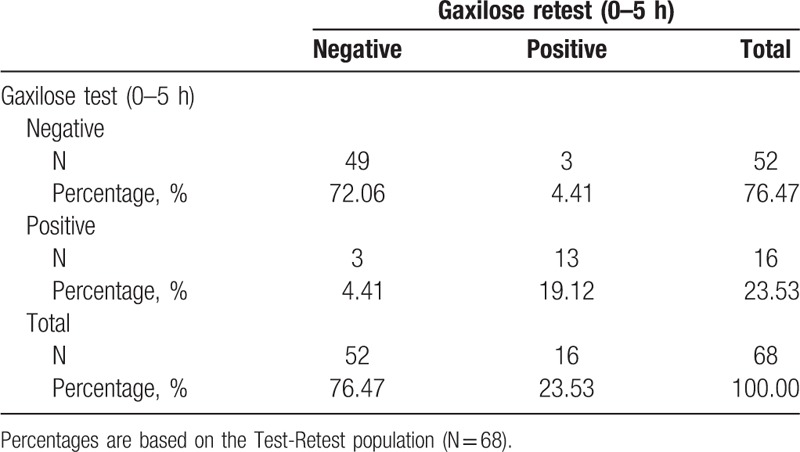
Results of the Gaxilose urine test and retest with urine accumulated from 0 to 5 hours (test–retest population).

### 3.6 Safety assessment

Among the overall population of the trial (N = 158), 148 patients (93.67%) were included in the safety population. Among them, 74 patients (50% of the safety population) were exposed to the GT and 74 patients (50% of the safety population) were exposed to the HBT.

Table 5 presents the summary of the AEs registered during the clinical trial. No serious adverse events (SAEs) or AEs of severe intensity were reported. A total number of 170 AEs were registered during the study in 83 patients (56.08%), all of mild or moderate intensity. Among these, 143 AEs, suffered by 70 patients (47.30%) were related to the GT or the HBT.

**Table 5:**
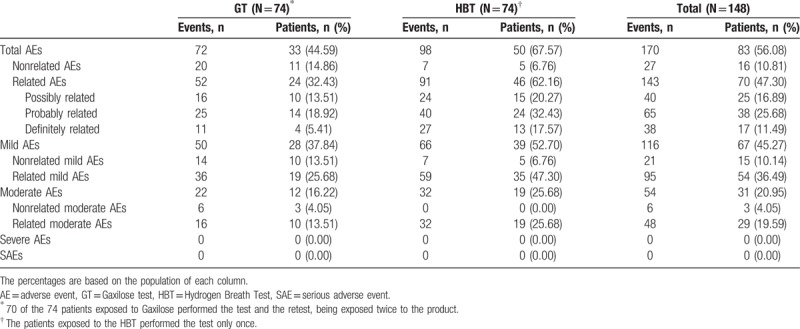
Summary of adverse events registered during the clinical trial (safety population).

The most frequent AEs were gastrointestinal disorders (143 among 170), specifically flatulence (28), abdominal distension (27), abdominal pain (26), nausea (20), and diarrhea (18). Gastrointestinal disorders were followed by nervous system disorders (20 among 170). Headache was the most frequently reported AE (14) in this group. The rest of AEs belonged to the group of respiratory, thoracic, and mediastinal disorders (3 among 170); skin and subcutaneous tissue disorders (2 among 170); renal and urinary disorders (1 among 170); and infections and infestations (1 among 170) (see Table 5, Supplemental Digital Content, which presents the complete list of AEs organized by system organ class).

Among the 74 patients exposed to Gaxilose, 24 patients (32.43%) presented 52 AEs related to Gaxilose, 36 mild and 16 moderate (Table 5). It must be taken into account that 70 of the 74 patients exposed to Gaxilose performed the test and the retest, being exposed twice to the product. Considering only the AEs that emerged the day of the first GT and in the 72 hours following it, 37 AEs related to Gaxilose (24 mild and 13 moderate) were reported for 22 patients (29.73% of the patients who performed the Gaxilose first test) (Table 6). The number of related AEs was higher for the 74 patients exposed to the HBT, as 91 AEs related to the test (59 mild and 32 moderate) were reported by 46 patients (62.16%) (Table 5).

**Table 6:**
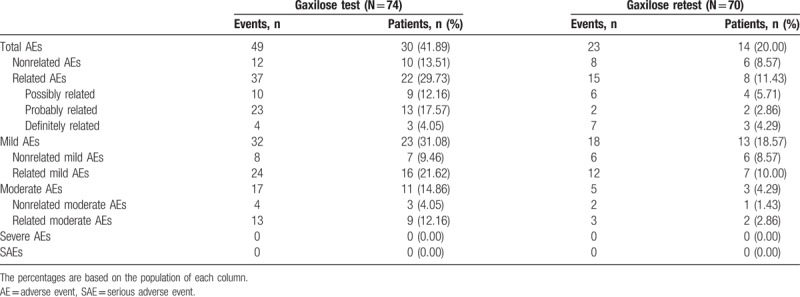
Summary of adverse events registered during the Gaxilose test and retest (safety population).

Finally, there was a significant decrease in the number of AEs and the number of patients affected in the Gaxilose retest in comparison with the first test. As it can be observed in Table 6, during the retest there were 15 AEs related to Gaxilose (12 mild and 3 moderate) in only 8 patients (11.43% of the patients who performed the Gaxilose retest).

## 4 Discussion

The results of this clinical trial have demonstrated the noninferiority of the GT compared to the HBT on the impact on diagnostic thinking for the diagnosis of hypolactasia. To complete the primary objective, we adopted a pretest/posttest approach, which is generally recommended and has been widely used to study the impact on diagnostic thinking of diagnostic agents.^[9–11,13,20,21]^ The difference between the impact on diagnostic thinking of the GT and the HBT was 7.46% (95% CI: 1.55%, infinite) for the ITT population and 7.77% (95% CI: 1.70%, infinite) for the PP population. The lower limits of the 95% CI are higher than the preestablished limit of noninferiority (−10%). Moreover, they are higher than 0, suggesting that the GT is in fact superior to the HBT on the impact on diagnostic thinking.^[22]^ It must also be pointed out that the results obtained for the ITT population and the PP population are almost identical. This fact is relevant in noninferiority clinical trials because many authors defend that errors in a trial conduct favor noninferiority.^[22]^ The similarity between the results of both populations is an indicator that the trial has been correctly designed, conducted, and monitored.

The secondary objective of this clinical trial was to demonstrate the noninferiority of the GT compared to the HBT on the impact on patient management. The pretest/posttest approach was also useful and has also been recommended for that purpose.^[9,10,13]^

The impact on patient management was evaluated through the percentage of patients who passed from “*No intervention*” or “*Prescription of additional diagnostic tests*” or “*Referral to another specialist*” at pretest to “*Diet adjustment and follow-up*” at posttest. In the case of the ITT population, only 5 patients (6.85%) of the 73 patients included in the GT arm and 4 patients (5.41%) of the 74 patients included in the HBT arm fulfilled this condition. In the case of the PP population, the number of patients was 4 (5.63%) among the 71 patients included in the GT arm and 4 (5.88%) among the 68 patients included in the HBT arm. The reasons behind these low numbers were related to 2 factors. First of all, as it can be seen in Tables 1 and 2 of Supplemental Digital Content, the option most frequently indicated in the pretest management questionnaire was “*Diet adjustment and follow-up*” (117 patients for the ITT population, 79.59%, and 111 patients for the PP population, 79.86%). This result should have been anticipated because the patients recruited in this clinical trial had to present clinical symptoms suggestive of lactose intolerance. The physician had to consider probable the diagnosis of hypolactasia, and therefore the adjustment of the diet would be the expected therapy for the majority of the patients. These patients were all ruled out for the calculation of the impact on patient management.

The second factor is related to the percentage of positive diagnoses of the study. The physician would change the expected therapy of a patient from “*No intervention*” or “*Prescription of additional diagnostic tests*” or “*Referral to another specialist*” at pretest to “*Diet adjustment and follow-up*” at posttest only if the result of the test indicates an hypolactasia diagnosis. However, the number of positive diagnoses was low: only 42 (28.57%) in the ITT population and 40 (28.78%) in the PP population. This percentage is lower than that found in the previous Gaxilose efficacy clinical trial, in which the positive diagnoses represented 53.2%, even though the inclusion and exclusion criteria were almost the same.^[8]^ A possible explanation could be that the number of patients with symptoms of lactose intolerance was low in the participating centers, and that this fact favored the recruitment of patients with a less clear symptomatology. Maybe, the number of participating centers should be higher in next clinical trials to compensate this possible bias.

In spite of this limitation, the noninferiority of the GT compared to the HBT on the impact on patient management was demonstrated, also with similar results for the ITT and PP populations of the trial. The difference between the impact of the GT and the HBT was 1.44% (95% CI: −6.31% to 9.20%) for the ITT population and 0.25% (95% CI: −8.00% to 7.50%) for the PP population.

Altogether, the results obtained demonstrate the noninferiority of the GT compared to the HBT on the impact on diagnostic thinking and patient management. Therefore, the clinical benefit of the GT has been proved, even more considering that the diagnostic accuracy of the GT was shown to be superior to that of the HBT, with significantly higher sensitivity and specificity values, in the previous phase IIb/III clinical trial.^[8]^ This study represents the first evaluation of the diagnostic and therapeutic impact of hypolactasia diagnostic methods.

The other secondary objectives were to demonstrate GT reproducibility with urine accumulated from 0 to 4 hours and from 0 to 5 hours after Gaxilose administration. The EMA guideline on Clinical Evaluation of Diagnostic Agents^[13]^ establishes that reproducibility assessments are more meaningful when they are performed in the same subject. For that purpose, the patients randomly assigned to the GT arm had to perform the test twice, following a test–retest reproducibility evaluation model.

The GT with urine accumulated from 0 to 4 hours is not commercialized, but its efficacy and safety were demonstrated in 3 clinical trials along with those of the GT with urine accumulated from 0 to 5 hours and those of the Gaxilose blood test. Its sensitivity and specificity were excellent, >90%.^[7,8]^ The aim of determining the test–retest reproducibility of this method was to gather information to evaluate the possibility of reducing the length of the GT 1 hour, and commercializing the test with urine accumulated from 0 to 4 hours.

The hypotheses preestablished in the trial protocol expected an agreement Kappa coefficient considered as at least substantial or almost perfect (>0.6)^[23]^ and an ICC >0.6.^[17,18]^ However, as shown in Table 3, the diagnoses of the test and retest were discrepant for 10 patients (14.71% of the test–retest population), the ICC was 0.5468, and the Kappa coefficient was found to be 0.5913 (95% CI: 0.3639–0.8188), indicating a moderate agreement between test and retest.^[23]^ In the case of the GT with urine accumulated from 0 to 5 hours, the diagnoses of the test and retest were discrepant for 6 patients (8.82% of the test–retest population) (Table 4), the ICC was 0.5761, but the Kappa coefficient was 0.7548 (95% CI: 0.5692–0.9404), indicating a substantial agreement between test and retest.^[23]^ In the light of these results, it can be concluded that the test–retest reproducibility is better for the GT with urine accumulated from 0 to 5 hours. Consequently, the possibility of reducing one hour the length of the urine collection period of the GT is currently ruled out. On the other hand, to our knowledge, the reproducibility of other lactose malabsorption diagnostic methods (biopsy, HBT, lactose tolerance test) has not been evaluated in the same subject and in a period of time as short as 5 ± 2 days. Therefore, the reproducibility of the Gaxilose tests cannot be compared with that of other diagnostic methods.

Regarding safety assessment, the most important result was that no SAEs were detected during the conduct of the study (Table 5), being all the 170 registered AEs of mild or moderate intensity. Considering only the first GT and the 72 hours following it, 37 AEs related to Gaxilose (24 mild and 13 moderate) were reported for 22 patients (29.73% of the patients who performed the first GT). These data represent a significant increase in the number of AEs and the percentage of patients affected when compared with previous Gaxilose clinical trials. No Gaxilose-related AEs were reported during the phase I and phase Ib clinical trials.^[7]^ During the phase IIb/III clinical trial, only 8 AEs in 5 participants among 205 (2.4%) in the urine test and 5 AEs in 4 participants among 203 (2.0%) in the blood test were reported as at least possibly related to Gaxilose.^[8]^ The reason underlying this significant increase in the number of AEs related to Gaxilose and the percentage of patients affected is unknown and cannot be explained. Nevertheless, the number of AEs reported and the number of affected patients was significantly lower during the Gaxilose retest (Table 6) and closer to the data of the previous phase IIb/III clinical trial: 15 AEs related to Gaxilose (12 mild and 3 moderate) in only 8 patients (11.43% of the patients who performed the Gaxilose retest). This suggests that part of the AEs observed during the first test could be due to the anxiety of performing an unknown procedure, disappearing during the retest.

Concerning the HBT arm, the number of related AEs was higher, since 91 AEs related to the test (59 mild and 32 moderate) were reported by 46 of the 74 patients exposed (62.16%). These results were expected because the HBT is known to produce abdominal discomfort and other AEs due to the high doses of lactose administrated to the patients,^[24]^ which in our study were comprised between 25 and 50 g of lactose.

As general conclusion, the results of this clinical trial have demonstrated the noninferiority of the GT compared to the HBT on the impact on diagnostic thinking and on patient management for the diagnosis of hypolactasia both for the ITT and PP populations of the study, proving the clinical benefit of the performance of the GT. The reproducibility of the GT with urine accumulated from 0 to 4 hours was not as good as expected because the hypothesis of an ICC >0.6 and of the agreement being considered as at least substantial was not verified. It must be reminded that this test is not currently commercialized. However, in the case of the GT with urine accumulated from 0 to 5 hours, even though the ICC was close to but <0.6, the Kappa coefficient was indicative of substantial agreement between the test and retest diagnoses.^[23]^ Finally, the GT can still be considered a safe diagnostic method because no SAEs were reported, and all the Gaxilose-related AEs were of mild or moderate intensity.

## Acknowledgments

The authors thank the participants in the different centers: Sonia Rodríguez and Miriam Menacho (Hospital Universitario Ramón y Cajal); Ana Godino and Dr. María Teresa Pérez (Hospital Universitario de la Princesa); Dr. Mariano Gómez-Rubio, Dr. Elena San Miguel, Dr. Guillermo Castillo, Dr. Tomás Pascual, Carmen Carrasco and María Antonia Morato (Hospital Universitario de Getafe); Rocío Gallardo and Estefanía Valencia (Hospital Universitario Virgen de la Victoria); Pilar Hallado and Ángel Estébanez (Hospital Universitario Marqués de Valdecilla); and Anna Bahí, Nuria Cle and Carla Coll (Hospital Universitario Doctor Josep Trueta). The authors also thank Emanuela Mollica for her contribution to the initial design of the study.

## Author contributions

**Conceptualization:** Carmen Monsalve-Hernando, Carmen Hermida.

**Formal analysis:** Carmen Monsalve-Hernando, Carmen Hermida.

**Investigation:** Laura Crespo, Blanca Ferreiro, Verónica Martín, Xavier Aldeguer, Verónica Opio, Pedro Luis Fernández-Gil, Eduardo Romero, Carmen Lara, Cecilio Santander, Leyanira Torrealba, Theodora Savescu.

**Methodology:** Carmen Monsalve-Hernando, Laura Crespo, Blanca Ferreiro, Verónica Martín, Xavier Aldeguer, Verónica Opio, Pedro Luis Fernández-Gil, María Jesús Gaspar, Eduardo Romero, Carmen Lara, Cecilio Santander, Leyanira Torrealba, Theodora Savescu, Carmen Hermida.

**Project administration:** Carmen Monsalve-Hernando, Laura Crespo, María Jesús Gaspar, Carmen Hermida.

**Resources:** Laura Crespo, Blanca Ferreiro, Verónica Martín, Xavier Aldeguer, Verónica Opio, Pedro Luis Fernández-Gil, María Jesús Gaspar, Cecilio Santander.

**Supervision:** Carmen Monsalve-Hernando, Laura Crespo, Blanca Ferreiro, Verónica Martín, Xavier Aldeguer, Verónica Opio, Pedro Luis Fernández-Gil, María Jesús Gaspar, Cecilio Santander, Carmen Hermida.

**Validation:** Carmen Monsalve-Hernando, María Jesús Gaspar, Carmen Hermida.

**Visualization:** Carmen Monsalve-Hernando, Carmen Hermida.

**Writing—original draft:** Carmen Monsalve-Hernando.

**Writing—review and editing:** Carmen Monsalve-Hernando, Laura Crespo, Blanca Ferreiro, Verónica Martín, Xavier Aldeguer, Verónica Opio, Pedro Luis Fernández-Gil, María Jesús Gaspar, Eduardo Romero, Carmen Lara, Cecilio Santander, Leyanira Torrealba, Theodora Savescu, Carmen Hermida.

Carmen Hermida orcid: 0000-0003-3337-647X.

## Supplementary Material

Supplemental Digital Content

## References

[1] Misselwitz B Pohl D Frühauf H Lactose malabsorption and intolerance: pathogenesis, diagnosis and treatment. United European Gastroenterol J 2013;1:151–9. 10.1177/2050640613484463 PMC4040760 24917953

[2] Mattar R de Campos Mazo DF Carrilho FJ Lactose intolerance: diagnosis, genetic, and clinical factors. Clin Exp Gastroenterol 2012;5:113–21. 22826639 10.2147/CEG.S32368 PMC3401057

[3] Vandenplas Y Lactose intolerance. Asia Pac J Clin Nutr 2015;24 Suppl. 1:S9–13. 26715083 10.6133/apjcn.2015.24.s1.02

[4] Hermida C Corrales G Martínez-Costa OH Noninvasive evaluation of intestinal lactase with 4-galactosylxylose: comparison with 3- and 2-galactosylxylose and optimization of the method in rats. Clin Chem 2006;52:270–7. 16384892 10.1373/clinchem.2005.058446

[5] Hermida C Corrales G Cañada FJ Optimizing the enzymatic synthesis of beta-d-galactopyranosyl-d-xyloses for their use in the evaluation of lactase activity in vivo. Bioorg Med Chem 2007;15:4836–40. 17512743 10.1016/j.bmc.2007.04.067

[6] Ohkohchi N Himukai M Igarashi Y Mechanism of d-xylose transport in human small intestine. J Pediatr Gastroenterol Nutr 1986;5:372–8. 3723257 10.1097/00005176-198605000-00006

[7] Hermida C Guerra P Martínez-Costa OH Phase I and phase IB clinical trials for the noninvasive evaluation of intestinal lactase with 4-galactosylxylose (gaxilose). J Clin Gastroenterol 2013;47:501–8. 23328304 10.1097/MCG.0b013e318272f507

[8] Aragón JJ Hermida C Martínez-Costa OH Noninvasive diagnosis of hypolactasia with 4-galactosylxylose (gaxilose): a multicentre, open-label, phase IIB-III nonrandomized trial. J Clin Gastroenterol 2014;48:29–36. 23722657 10.1097/MCG.0b013e318297fb10

[9] Fryback DG Thornbury JR The efficacy of diagnostic imaging. Med Decis Making 1991;11:88–94. 1907710 10.1177/0272989X9101100203

[10] Dixon AK The impact of medical imaging on the physician's diagnostic and therapeutic thinking. Eur Radiol 1998;8:488–90. 9510594 10.1007/s003300050423

[11] Van den Bruel A Cleemput I Aertgeerts B The evaluation of diagnostic tests: evidence on technical and diagnostic accuracy, impact on patient outcome and cost-effectiveness is needed. J Clin Epidemiol 2007;60:1116–22. 17938052 10.1016/j.jclinepi.2007.03.015

[12] Lijmer JG Leeflang M Bossuyt PMM Proposals for a phased evaluation of medical tests. Med Decis Making 2009;29:E13–21. 19605881 10.1177/0272989X09336144

[13] European Medicines Agency. Committee for Medicinal Products for Human Use. Guideline on clinical evaluation of diagnostic agents. (CPMP/EWP/1119/98/Rev.1.) 2009.

[14] Jellema P Schellevis FG van der Windt DA Lactose malabsorption and intolerance: a systematic review on the diagnostic value of gastrointestinal symptoms and self-reported milk intolerance. QJM 2010;103:555–72. 20522486 10.1093/qjmed/hcq082

[15] LacTEST 0.45 g Summary of product characteristics. Available at: http://www.venterpharma.com/lactest/ Accessed May 22, 2018.

[16] Hermida C Martínez-Costa OH Corrales G Improvement and validation of d-xylose determination in urine and serum as a new tool for the noninvasive evaluation of lactase activity in humans. J Clin Lab Anal 2014;28:478–86. 24659338 10.1002/jcla.21713 PMC6807429

[17] Chinn S Statistics in respiratory medicine. 2. Repeatability and method comparison. Thorax 1991;46:454–6. 1858087 10.1136/thx.46.6.454 PMC463197

[18] Fleiss JL Levin B Cho Paik M Statistical Methods for Rates and Proportions. 3rd ed. Hoboken, NJ: John Wiley & sons; 2003.

[19] McHugh ML Interrater reliability: the kappa statistic. Biochem Med (Zagreb) 2012;22:276–82. 23092060 PMC3900052

[20] Hobby JL Dixon AK Bearcroft PW MR imaging of the wrist: effect on clinical diagnosis and patient care. Radiology 2001;220:589–93. 11526253 10.1148/radiol.2203001429

[21] Ashby E Roposch A Diagnostic yield of sonography in infants with suspected hip dysplasia: diagnostic thinking efficiency and therapeutic efficiency. AJR Am J Roentgenol 2015;204:177–81. 25539254 10.2214/AJR.14.12477

[22] Schumi J Wittes JT Through the looking glass: understanding non-inferiority. Trials 2011;12:106. 21539749 10.1186/1745-6215-12-106 PMC3113981

[23] Landis JR Koch GG The measurement of observer agreement for categorical data. Biometrics 1977;33:159–74. 843571

[24] Gasbarrini A Corazza GR Gasbarrini G Methodology and indications of H2-breath testing in gastrointestinal diseases: the Rome Consensus Conference. Aliment Pharmacol Ther 2009;29 Suppl 1:1–49. 19344474 10.1111/j.1365-2036.2009.03951.x

