# Supplementary material for: Phase IV noninferiority controlled randomized trial to evaluate the impact on diagnostic thinking and patient management and the test–retest reproducibility of the Gaxilose test for hypolactasia diagnosis
Source: Medicine (Baltimore). 2018 Nov 16;97(46):e13136. doi: 10.1097/MD.0000000000013136 (PMC6257416; doi:10.1097/MD.0000000000013136)
Supplement: Supplemental Digital Content [file medi-97-e13136-s001.pdf]

SUPPLEMENTAL DIGITAL CONTENT

Phase IV non-inferiority controlled randomized trial to evaluate the impact on diagnostic thinking and on patient management and the test retest reproducibility of the Gaxilose test for hypolactasia diagnosis.

Carmen Monsalve-Hernando, PhD, Laura Crespo, MD, PhD, Blanca Ferreiro, MD, Verónica Martín, MD, Xavier Aldeguer, MD, PhD, Verónica Opio, MD, Pedro Luis Fernández-Gil, MD, María Jesús Gaspar, PhD, Eduardo Romero, MD, Carmen Lara, MD, Cecilio Santander, MD, PhD, Leyanira Torrealba, MD, PhD, Theodora Savescu, MD, Carmen Hermida, PhD.

Code: **VPH-GXL-2013-01** Centre: ______________________

Sponsor**: Venter Pharma S.L.** Patient number: ______________________

Questionnaire to assess the impact on patient management

PRE-TEST QUESTIONNAIRE

- *Indicate your predicted patient management, before the diagnostic test is performed (select only one option among those suggested):*

☐ *No intervention*

☐ *Diet adjustment and follow- up*

☐ *Prescribe genetic test for primary hypolactasia*

☐ *Prescribe diagnostic tests for celiac disease*

☐ *Prescribe diagnostic tests for Crohn disease*

☐ *Prescribe diagnostic tests for cystic fibrosis*

☐ *Other diagnostic tests*

☐ *Referral to Oncologist*

☐ *Referral to Endocrinologist*

☐ *Referral to Rheumatologist*

☐ *Referral to Nutritionist*

☐ *Referral to another specialist*

Figure 1. Example of the pre-test management questionnaire used during the clinical trial. The options represent all the available therapeutic management strategies and are the same in the post-test management questionnaire.

Table 1. Results of the pre-test and post-test intended management questionnaires (ITT population).

	ITT Population (N = 147)		
	**GT (N = 73)**	**HBT (N = 74)**	**Total (N = 147)**
**Pre-test management questionnaire (n (%))**			
No intervention	10 (13.70)	6 (8.11)	16 (10.88)
Diet adjustment and follow up	55 (75.34)	62 (83.78)	117 (79.59)
Diagnostic test for celiac disease	2 (2.74)	0 (0.00)	2 (1.36)
Other diagnostic tests	5 (6.85)	6 (8.11)	11 (7.48)
Diagnostic test for Crohn's disease	1 (1.37)	0 (0.00)	1 (0.68)
**Post-test management questionnaire (n (%))**			
No intervention	21 (28.77)	20 (27.03)	41 (27.89)
Referral to a nutritionist	1 (1.37)	0 (0.00)	1 (0.68)
Referral to another specialist	1 (1.37)	1 (1.35)	2 (1.36)
Diet adjustment and follow up	32 (43.84)	34 (45.95)	66 (44.90)
Diagnostic test for celiac disease	3 (4.11)	1 (1.35)	4 (2.72)
Other diagnostic tests	13 (17.81)	18 (24.32)	31 (21.09)
Diagnostic test for Crohn's disease	2 (2.74)	0 (0.00)	2 (1.36)

Percentages are based on the population of each column.

GT: Gaxilose test; HBT: Hydrogen Breath Test; ITT: Intent-to-treat.

Table 2. Results of the pre-test and post-test intended management questionnaires (PP population).

	**PP Population (N = 139)**		
	**LacTEST (N = 71)**	**HBT (N = 68)**	**Total (N = 139)**
**Pre-test management questionnaire (n (%))**			
No intervention	10 (14.08)	6 (8.82)	16 (11.51)
Diet adjustment and follow up	54 (76.06)	57 (83.82)	111 (79.86)
Diagnostic test for celiac disease	2 (2.82)	0 (0.00)	2 (1.44)
Other diagnostic tests	4 (5.63)	5 (7.35)	9 (6.47)
Diagnostic test for Crohn's disease	1 (1.41)	0 (0.00)	1 (0.72)
**Post-test management questionnaire (n (%))**			
No intervention	21 (29.58)	15 (22.06)	36 (25.90)
Referral to a nutritionist	1 (1.41)	0 (0.00)	1 (0.72)
Referral to another specialist	1 (1.41)	1 (1.47)	2 (1.44)
Diet adjustment and follow up	30 (42.25)	34 (50.00)	64 (46.04)
Diagnostic test for celiac disease	3 (4.23)	1 (1.47)	4 (2.88)
Other diagnostic tests	13 (18.31)	17 (25.00)	30 (21.58)
Diagnostic test for Crohn's disease	2 (2.82)	0 (0.00)	2 (1.44)

Percentages are based on the population of each column.

GT: Gaxilose test; HBT: Hydrogen Breath Test; PP: Per Protocol.

Table 3. Discrepant results between Gaxilose test and retest with urine accumulated from 0-4 hours.

Patient	Test (0-4 h)			Retest (0-4 h)		
	Urine V (mL)	Total amount of xylose (mg)	Diagnosis	Urine V (mL)	Total amount of xylose (mg)	Diagnosis
0113	270	16.64	Hypolactasia	450	46.38	Normolactasia
0122	860	54.79	Normolactasia	410	21.55	Hypolactasia
0133	350	31.48	Normolactasia	320	25.57	Hypolactasia
0216	265	30.17	Normolactasia	410	27.37	Hypolactasia
0419	450	20.08	Hypolactasia	260	37.69	Normolactasia
0612	850	45.29	Normolactasia	600	23.03	Hypolactasia
0615	250	15.15	Hypolactasia	200	64.06	Normolactasia
0618	150	24.62	Hypolactasia	150	29.85	Normolactasia
0620	100	14.59	Hypolactasia	350	29.29	Normolactasia
0621	200	49.39	Normolactasia	50	22.47	Hypolactasia

Table 4. Discrepant results between Gaxilose test and retest with urine accumulated from 0-5 hours.

Patient	Test (0-5 h)			Retest (0-5 h)		
	Urine V (mL)	Total amount of xylose (mg)	Diagnosis	Urine V (mL)	Total amount of xylose (mg)	Diagnosis
0122	954	60.89	Normolactasia	550	27.10	Hypolactasia
0138	260	51.34	Normolactasia	80	34.68	Hypolactasia
0220	145.1	34.01	Hypolactasia	341	41.69	Normolactasia
0419	710	36.55	Hypolactasia	374	54.33	Normolactasia
0612	1000	58.89	Normolactasia	800	34.37	Hypolactasia
0615	540	27.32	Hypolactasia	300	75.07	Normolactasia

Table 5. Adverse events reported during the clinical trial organized by system organ class.

	GT (N = 72)		HBT (N = 98)		Total (N = 170)	
	**n**	**%**	**n**	**%**	**n**	**%**
**Gastrointestinal disorders**						
Flatulence	6	8.33	22	22.44	28	16.47
Abdominal distensión	14	19.44	13	13.27	27	15.88
Abdominal pain	16	22.22	10	10.20	26	15.29
Nausea	10	13.89	10	10.20	20	11.76
Diarrhoea	5	6.94	13	13.27	18	10.59
Abdominal pain upper	2	2.78	4	4.08	6	3.53
Abdominal discomfort	2	2.78	3	3.06	5	2.94
Gastrointestinal sounds abnormal	1	1.39	3	3.06	4	2.35
Vomiting	2	2.78	1	1.02	3	1.76
Dyspepsia	0	0.00	2	2.04	2	1.18
Gastroesophageal reflux disease	0	0.00	1	1.02	1	0.59
Dysphagia	0	0.00	1	1.02	1	0.59
Regurgitation	0	0.00	1	1.02	1	0.59
Eructation	0	0.00	1	1.02	1	0.59
**Nervous System Disorders**						
Headache	9	12.50	5	5.10	14	8.24
Dizziness	1	1.39	3	3.06	4	2.35
Syncope	1	1.39	0	0.00	1	0.59
Paraesthesia	0	0.00	1	1.02	1	0.59
**Respiratory, thoracic and mediastinal disorders**						
Oropharyngeal pain	1	1.39	0	0.00	1	0.59
Throat irritation	0	0.00	1	1.02	1	0.59
Oropharyngeal discomfort	0	0.00	1	1.02	1	0.59
**Skin and subcutaneous tissue disorders**						
Rash	1	1.39	1	1.02	2	1.18
**Renal and urinary disorders**						
Urinary incontinence	1	1.39	0	0.00	1	0.59
**Infections and infestations**						
Nasopharyngitis	0	0.00	1	1.02	1	0.59

Percentages are based on the population of each column.

GT: Gaxilose test; HBT: Hydrogen Breath Test.
